# Monolayer autoxidation of arachidonic acid to epoxyeicosatrienoic acids as a model of their potential formation in cell membranes

**DOI:** 10.1016/j.jlr.2021.100159

**Published:** 2021-12-02

**Authors:** James A. Weiny, William E. Boeglin, M. Wade Calcutt, Donald F. Stec, Alan R. Brash

**Affiliations:** 1 Department of Pharmacology, Vanderbilt University, Nashville, TN, USA; 2 The Vanderbilt Institute of Chemical Biology, Vanderbilt University, Nashville, TN, USA; 3 Department of Biochemistry, Vanderbilt University, Nashville, TN, USA; 4 Department of Chemistry, Vanderbilt University, Nashville, TN, USA

**Keywords:** EET, epoxide, eicosanoids, HETE, δ-lactone, lipid biochemistry, liquid chromatography, proton NMR, EET, epoxyeicosatrienoic acid, H(P)ETE, hydro(pero)xyeicosatetraenoic acid, RP-HPLC, reversed-phase HPLC, SP-HPLC, straight phase HPLC

## Abstract

In light of the importance of epoxyeicosatrienoic acids (EETs) in mammalian pathophysiology, a nonenzymatic route that might form these monoepoxides in cells is of significant interest. In the late 1970s, a simple system of arranging linoleic acid molecules on a monolayer on silica was devised and shown to yield monoepoxides as the main autoxidation products. Here, we investigated this system with arachidonic acid and characterized the primary products. By the early stages of autoxidation (∼10% conversion of arachidonic acid), the major products detected by LC-MS and HPLC-UV were the 14,15-, 11,12-, and 8,9-EETs, with the 5,6-EET mainly represented as the 5-δ-lactone-6-hydroxyeicosatrienoate as established by ^1^H-NMR. The EETs were mainly the *cis* epoxides as expected, with minor trans configuration EETs among the products. ^1^H-NMR analysis in four deuterated solvents helped clarify the epoxide configurations. EET formation in monolayers involves intermolecular reaction with a fatty acid peroxyl radical, producing the EET and leaving an incipient and more reactive alkoxyl radical, which in turn gives rise to epoxy-hydro(pero)xides and other polar products. The monolayer alignment of fatty acid molecules resembles the arrangements of fatty acids in cell membranes and, under conditions of lipid peroxidation, this intermolecular mechanism might contribute to EET formation in biological membranes.

In the late 1970s, James F. Mead *et al.* reported on the products from a simple and ingenious set-up for conducting linoleic acid autoxidation with the fatty acid arranged in a molecular monolayer (1). This was designed to mimic the arrangement of esterified lipids in a biological membrane. By soaking chromatographic silica with a hexane solution of linoleic acid after decanting away the solvent and drying the silica, this resulted in a surface coating of the fatty acid as a molecular monolayer (2). Autoxidation of this linoleic acid on silica with subsequent analysis by TLC and GC-MS identified prominent fatty acid monoepoxides as products, with the 9,10- and 12,13-epoxides in similar amounts (1). By comparison, in a conventional bulk phase or solution autoxidation, the main primary products were fatty acid hydroperoxides with no trace of monoepoxides (1), as is well established (3, 4).

These observations were followed up over the years, by Mead, Sevanian, and others (5, 6, 7, 8). These included in vitro autoxidation experiments with soybean phosphatidylcholine liposomes, from which multiple linoleate-derived monoepoxides, hydroperoxides, and more polar derivatives were identified (5). Also, from an in vivo experiment, the content of esterified linoleate epoxides in lung tissues was enhanced in live rats breathing an atmosphere containing 6.5 ppm nitrogen dioxide (6) and attributed at least partly to monolayer-type autoxidation in the cell membranes.

With the now long-standing interest in the biosynthesis and biological activities of the monoepoxides of arachidonic acid, (epoxyeicosatrienoic acids, EETs) (e.g., refs (9, 10, 11, 12, 13, 14, 15)), and their occurrence esterified in tissue phospholipids (16, 17, 18, 19, 20), it was of interest to carry out the monolayer autoxidation on silica as a potential mimic of what might occur in vivo and potentially contribute to the production of EETs in biological membranes.

## Materials and methods

### Materials

The arachidonic acid used in these experiments was purchased from Nuchek Prep (Elysian, MN). Silica Gel 60 G (EM Reagents), listed as containing CaSO4 13%, Cl 0.02%, and Fe 0.02%, was placed in a covered Pyrex cell culture dish and oven dried overnight at 130°C for 24 h before use in the autoxidation experiments. Standards of EETs were prepared by epoxidation of arachidonic acid using an equivalent of meta-chloroperoxybenzoic acid in dichloromethane.

### Monolayer autoxidation experiments

Arachidonic acid (50 mg) was dissolved in 4 ml hexane in a 5 ml glass Reacti-Vial, and 200 mg of dried silica was added. After gentle mixing for at least 10 min under argon, the hexane was fully decanted and collected for subsequent analysis of the remaining arachidonic acid, assayed in comparison to aliquots of the pure 50 mg/4 ml hexane solution by quantitative UV analysis of aliquots converted to 15-hydro(pero)xyeicosatetraenoic acid (HPETE) (ε 25,000 at 236 nm) using soybean lipoxygenase (21). The silica exposed to arachidonic acid was dried carefully under a gentle stream of nitrogen, and with the Reacti-Vial held sideways, the silica was distributed as an even layer along the length of the vial. The monolayer autoxidations were conducted with the uncapped silica-containing vials placed sideways in a 60°C oven for 45 min, or in a 37°C incubator for 6 h in ambient air. These suitable times for approximately 10% oxidation of the arachidonic acid were established empirically by examining several reaction conditions. For comparison, in the original work by Mead *et al.*, the monolayers of linoleic acid were 25–30% oxidized in 1 h at 60°C (1). Upon completion, the vials were promptly cooled on ice and the silica extracted using 4 ml MeOH and aliquots used for initial HPLC-UV analysis.

### LC-MS analyses

LC-MS profiles of monolayer autoxidations and EET standards were analyzed using a Thermo TSQ Vantage Triple Quadrapole MS instrument (Thermo Fisher Scientific, Waltham, MA). Reversed-phase HPLC (RP-HPLC) analysis was performed with electrospray ionization in the negative ion mode. A Phenomenex C18 5 μ column (25 × 0.46 cm) was eluted isocratically with acetonitrile/water/glacial acetic acid (80:20:0.01 by volume) at a flow rate of 1 ml/min. The electrospray voltage was set at 4.0 kV: vaporizer temperature at 300°C; sheath and auxiliary gas pressure at 50 and 5 ψ, respectively; and capillary temperature at 300°C.

### HPLC-UV analyses

Aliquots of the monolayer autoxidations were analyzed by RP-HPLC using a Waters Symmetry C18 column (25 × 0.46 cm), with a solvent of acetonitrile/water/glacial acetic acid (75/25/0.01 or 70/30/0.01 by volume), at a flow rate of 1 ml/min, with on-line UV detection (Agilent 1100 series diode array detector). Further identification in comparison to EET standards was achieved by straight phase HPLC (SP-HPLC) using a Thomson Advantage 5 μ silica column (25 × 0.46 cm) using a solvent of hexane/isopropanol/glacial acetic acid (100/0.5/0.02, by volume) run at 1 ml/min. For purification of the methyl ester of 14,15-*cis*-EET before NMR (designated as EET-2 in Results), the SP-HPLC column was eluted with hexane/isopropanol 100/0.5 by volume (retention time 8.2 min at 0.5 ml/min); the methyl ester of 14,15-*trans*-EET (EET-3 in Results) was purified by RP-HPLC using a solvent of acetonitrile/water 90:10 (retention time 21 min at 0.5 ml/min); and for the 5-δ-lactone-6-hydroxy derivative of 5,6-EET, the solvent used was 100/5/0.02 hexane/isopropanol/glacial acetic acid (retention time ∼ 24 min at 0.5 ml/min).

### NMR analyses

^1^H NMR and ^1^H,^1^H COSY NMR experiments were acquired using a 14.0 T Bruker magnet equipped with a Bruker AV-III console operating at 600.13 MHz. All the spectra were acquired in 3 mm NMR tubes using a Bruker 5 mm TCI cryogenically cooled NMR probe. The chemical shifts were referenced internally to benzene-d_6_ (7.16 ppm), and for the other three solvents, CDCl_3_ (7.26 ppm), d_3_-acetonitrile (1.93 ppm), and d_4_-methanol (3.30 ppm). For ∼100 μg of epoxide methyl ester, typical experimental conditions included 32K data points, 13 ppm sweep width, and a recycle delay of 1.5 s and 64 scans. For 2D 1H–1H COSY, the experimental conditions included 2,048 × 512 data matrix, 13 ppm sweep width, and a recycle delay of 1.5 s and four scans per increment. The data was processed using squared sinebell window function, symmetrized, and displayed in magnitude mode.

### GC-MS analyses

For unambiguous identification of individual EETs by GC-MS, the epoxides were acid hydrolyzed to the dihydroxy derivative and analyzed as the methyl ester trimethylsilyl ether derivative. EETs (approx. 5–10 μg) were treated with dimethoxyethane/water/70% perchloric acid (1:1:0.3 by volume) by first dissolving in 10 μl dimethoxyethane after addition of 13 μl of a 1:0.3 ratio solution of water and 70% perchloric acid (22). After reacting for 1 h at room temperature, the samples were dissolved in 0.5 ml DCM and washed with water until the pH readings matched that of deionized water (∼pH 5). After evaporating the solvent under N_2_ and subsequent derivatization to the methyl ester (diazomethane) and TMS ether (BSTFA), the aliquots of ∼100 ng were analyzed by GC-MS using a DB-5 column (30 m × 0.25 mm, Agilent) on a Thermo-Finnigan DSQ mass spectrometer operated in the positive ion electron impact mode (70 eV).

## Results

**Fig. 1:**
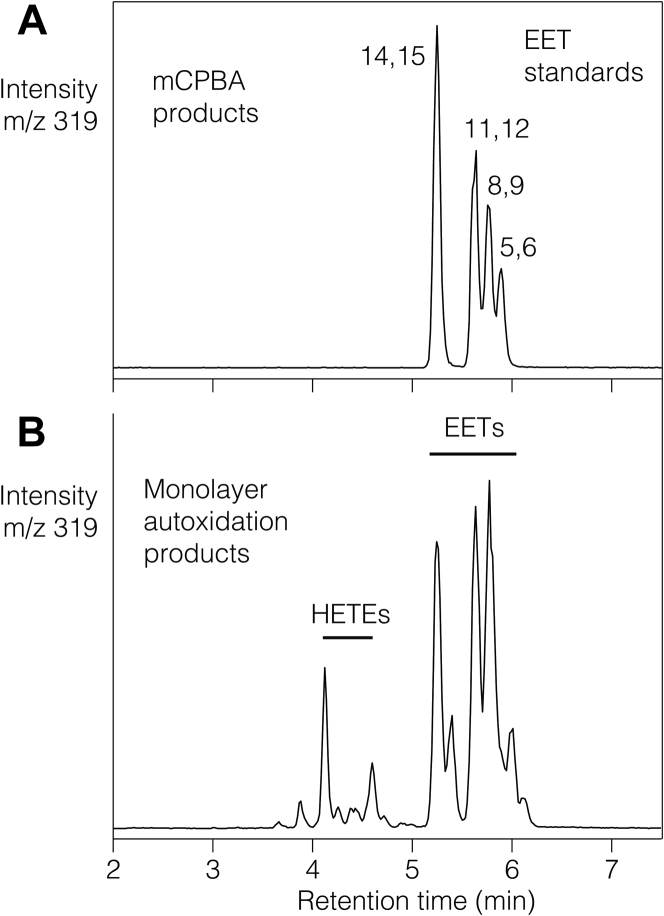
LC-MS analysis of EET standards and products of monolayer oxidation of arachidonic acid. A: The RP-HPLC was run with a solvent of acetonitrile/water/glacial acetic acid (80:20:0.01, by volume) at 1 ml/min with the EET standards (mCPBA products) detected as the [M-H]− anions at m/z 319. B: An aliquot from a monolayer autoxidation of arachidonic acid (60°C, 45 min) detects the [M-H]− ions representing the small peaks of HETEs after the more prominent peaks of EETs and EET isomers. Column: Waters 5 μ Symmetry C18 (25 × 0.46 cm); solvent acetonitrile/water/glacial acetic acid (80:20:0.01 by volume), with a flow rate of 1 ml/min. EET, epoxyeicosatrienoic acid; HETE, hydroxyeicosatetraenoic acid; mCPBA, meta-chloroperoxybenzoic acid; RP-HPLC, reversed phase-HPLC.

After mixing 50 mg arachidonic acid with 200 mg chromatographic silica in 4 ml hexane then decanting the solvent, the arachidonate absorbed on the silica was estimated as 38–40 mg as determined from subsequent assay of the excess remaining in the hexane solution. After drying the silica/arachidonic acid and conducting the monolayer autoxidations in ambient air at 60°C or 37°C, aliquots of the reactions were analyzed by LC-MS. As illustrated in Fig. 1, a preliminary analysis using relatively short retention times on reversed-phase LC-MS showed a profile of EET products detected as the [M-1]^−^ ions at m/z 319 that matched the retention times of a mixture of the four authentic standards, known to elute in the order 14,15-, 11,12-, 8,9-, and 5,6-EETs (23, 24). From the monolayer autoxidation, the peak corresponding to 14,15-EET and the small extra peak immediately after it at 5.3 min gave characteristic product ions of m/z 175 and 219 diagnostic for a 14,15-epoxide (25, 26, 27, 28). Similarly, 11,12-EET (product ions m/z 167 and 179) and 8,9-EET (m/z 155, 167 and 179) were identified, with the ions for 5,6-EET less abundant as became clear from further results below. In addition to EET-related products in the monolayer autoxidation sample, the earlier eluting small peaks at retention times of 4.5 and 4.9 min represented the [M-1]^−^ ions of 15-HETE and 5-HETE, respectively. These results provided evidence for the production of EETs in the monolayer autoxidation and their relative prominence over HETEs.

**Fig. 2:**
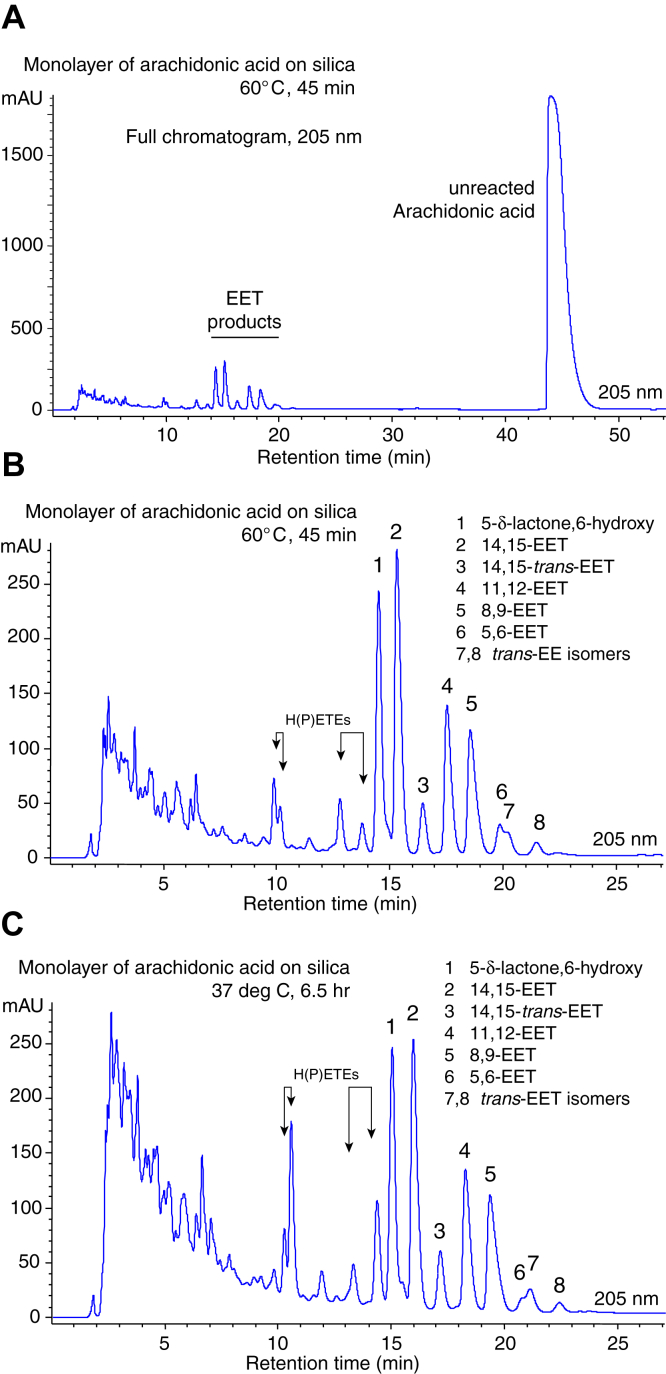
HPLC-UV analysis of products of the monolayer oxidation of arachidonic acid. A: The full RP-HPLC profile (205 nm) from a representative monolayer/silica autoxidation run at 60°C for 45 min shows the unreacted arachidonic acid eluting at 45 min and slightly saturated in the UV (1,750 mAU at 205 nm) and hence is even more dominant over the polar oxidation products or which the EET peaks are the most visible. Column: Waters 5 μ Symmetry C18 (25 × 0.46 cm); solvent acetonitrile/water/glacial acetic acid (70:30:0.01 by volume), with a flow rate of 1 ml/min with UV detection at multiple wavelengths and illustrated at 205 nm. B: A detailed look at the polar region of the same RP-HPLC chromatogram (205 nm). The four small arrows at 10–14 min denote the H(P)ETEs, in order of elution 15-HETE, 15-HPETE, 5-HETE, and 5-HPETE. The EET-related peaks are numbered EET-1 to EET-8. The identifications are based on matching retention times with authentic EET standards of 14,15-, 11,12-, 8,9, and 5,6-EETs, which additional evidence provided in later figures and the main text. C: A similar RP-HPLC analysis of polar products from a monolayer autoxidation run at 37°C for 6.5 h. The profiles match well to the results in panel B, with slightly more prominent peaks of H(P)ETEs and the same major and minor peaks of EETs. EET, epoxyeicosatrienoic acid; H(P)ETE, hydro(pero)xyeicosatetraenoic acid; RP-HPLC, reversed phase-HPLC.

**Fig. 3:**
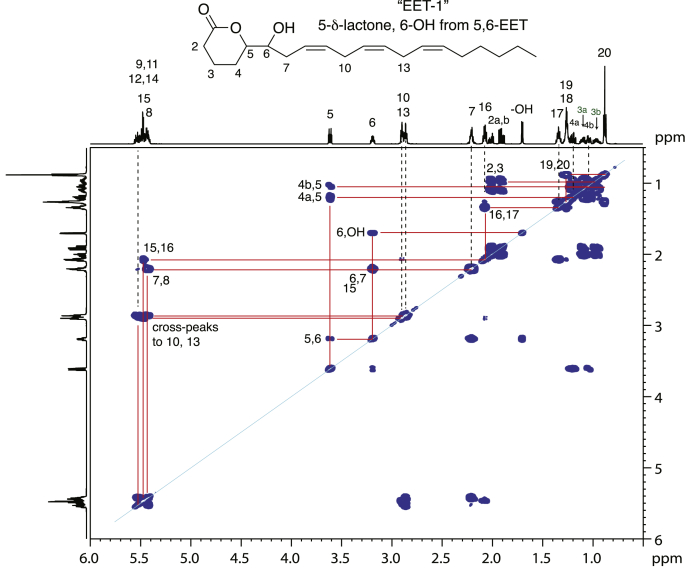
Proton NMR and COSY analysis of EET-1 (5-δ-lactone-6-hydroxy-DHET) in d_6_-benzene. EET-1 collected from larger amounts injected on RP-HPLC was further purified by SP-HPLC (HPLC-UV Methods) and the ^1^H-NMR spectrum and COSY correlation recorded in d_6_-benzene. Of significant interest, there are separate chemical shifts for the signals of H2, H3, and H4, a reflection of the chiral environment in the δ-lactone structure of EET-1. Other discernible chemical shifts are indicated on the figure, thus identifying EET-1 as 5-δ-lactone-6-hydroxy-eicosatrienoate. The spectral data are summarized in supplemental Table S1. DHET, dihydroxyeicosatrienoic acid; EET, epoxyeicosatrienoic acid; H(P)ETE, hydro(pero)xyeicosatetraenoic acid; RP-HPLC, reversed-phase HPLC; SP-HPLC straight phase HPLC.

A more detailed analysis and isolation of the individual EET-related products was conducted with isocratic elution at longer retention times on RP-HPLC and with UV detection at 205 nm, 220 nm, 235 nm, and 270 nm. As evident from the UV profile at 205 nm, Fig. 2A, the extent of autoxidation in this 60°C/45 min sample was around 10% as judged by the prominent peak of unreacted arachidonic acid compared with the combined peak areas of the polar oxidation products. The polar region of the same chromatogram is illustrated in Fig. 2B, and a similar chromatogram from a 37°C/6.5 h autoxidation in Fig. 2C. What had appeared at shorter retention times as a large peak of 14,15-EET was resolved into two approximately equal sized peaks in the better resolving RP-HPLC system. By comparison with the retention time of standards and later confirmed by GC-MS and NMR, 14,15-EET represents the second of the pair of peaks and is labeled as peak 2. The first major peak, designated as EET-1, was identified by NMR as the δ-lactone derivative of 5,6-EET, Fig. 3 (This internal ester was undetectable in the electrospray LC-MS analysis in Fig. 1 as it is does not produce carboxylate anions). As expected, treatment of the δ-lactone derivative with triethylamine, as described (29), produced the 5,6-dihydroxy derivative as a more polar peak on RP-HPLC (data not shown). The prominent peaks 4 and 5 corresponded to 11,12-EET and 8,9-EET, respectively, and were not subjected to further analysis. Based on HPLC retention times, the peak 6 represents a small peak of 5,6-EET, whose relative abundance is mostly accounted for as the δ-lactone derivative, EET-1. Notably, there was no significant formation of dihydroxyeicosatrienoic acids, the diol derivatives of EETs, in these autoxidation experiments.

**Fig. 4:**
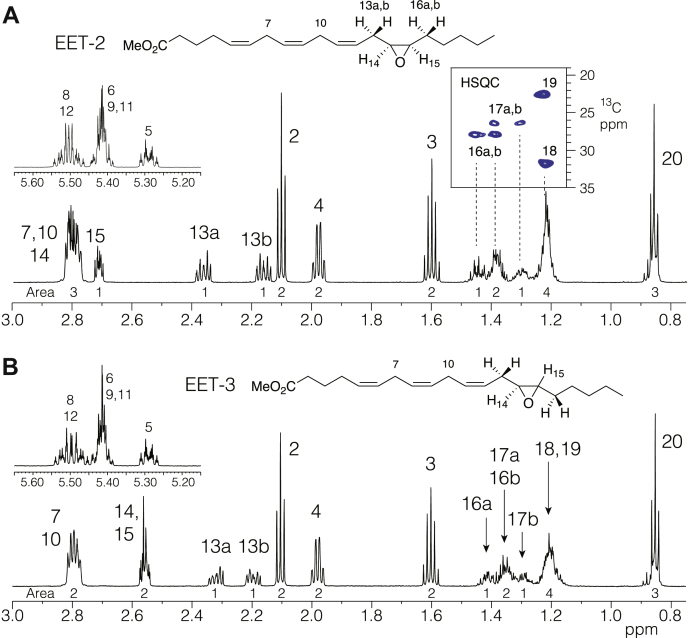
Proton NMR spectra of EET-2 (*cis*-14,15-EET) and EET-3 (*trans*-14,15-EET) methyl esters in d_6_-benzene. A: Spectrum of EET-2 methyl ester. The double bond region is shown in the top left inset. The methyl ester singlet at 3.345 ppm is the only signal not illustrated. Although the autoxidation product is racemic, the chemical structure of 14,15-EET is drawn as chiral to indicate the arrangement of protons around the epoxide. COSY analysis (not shown) helped with assignment of protons; HSQC analysis (inset, top right) defined the H16, 17, 18, and 19 multiplets at 1.45–1.8 ppm. B: Spectrum of EET-3 methyl ester. The double bond region is shown in the inset. The methyl ester singlet at 3.345 ppm is the only signal not illustrated. The spectra are tabulated in supplemental Table S2. EET, epoxyeicosatrienoic acid; HSQC, heteronuclear single quantum coherence.

**Fig. 5:**
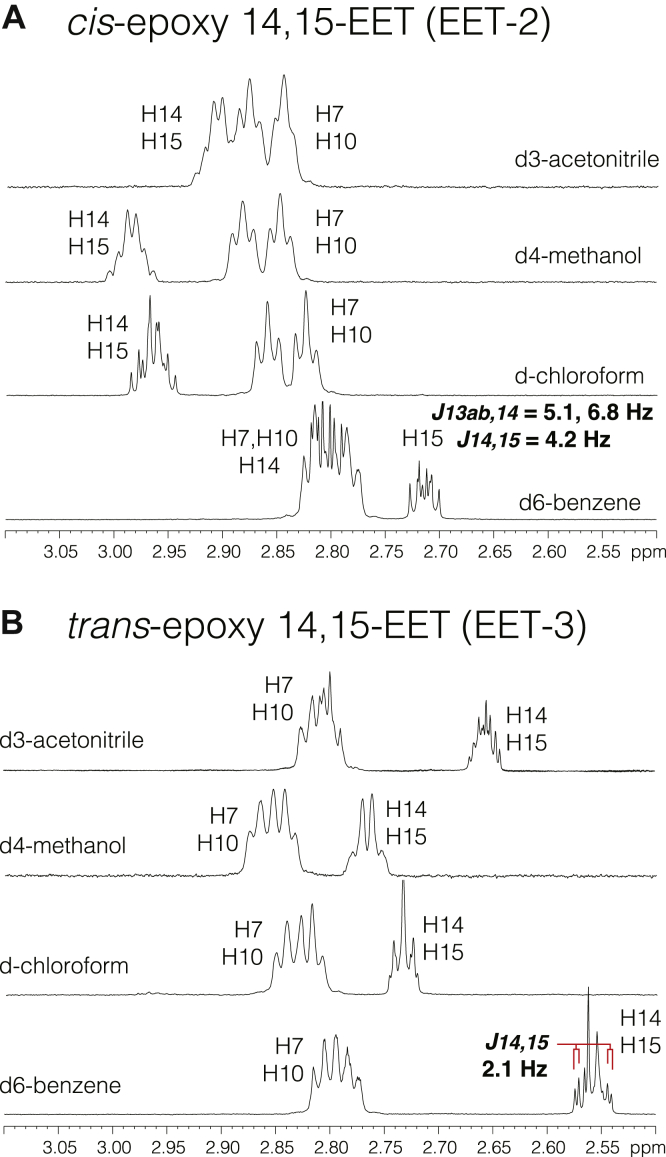
Proton NMR of the epoxide signals of EET-2 (*cis*-14,15-EET) and EET-3 (*trans*-14,15-EET) in four deuterated solvents. A: Chemical shifts (2.5–3.1 ppm) and multiplet patterns of EET-2 methyl ester recorded in (from top to bottom), d_3_-acetonitrile, d_4_-methanol, d-chloroform, and d_6_-benzene. Only in d_6_-benzene, H15 stands apart and appears as a double doublet of doublets (ddd), which with homonuclear decoupling of H16ab (not shown) clearly identifies a 4.2 Hz coupling for *J*_*14,15*_, thus establishing the 14,15-*cis*-epoxy configuration. B: A similar comparison conducted with EET-3 methyl ester also identified d_6_-benzene as the best solvent for discerning the *cis* or *trans* epoxide configuration. Although H14 and H15 overlap in d_6_-benzene, the 2.1 Hz coupling of each proton is evident at the sides of the multiplet, as confirmed as homonuclear decoupling of H13ab and H16ab, establishing the 14,15-*trans*-epoxy configuration. EET, epoxyeicosatrienoic acid.

By comparison with the four EET authentic standards, it was apparent both from the LC-MS and better resolving HPLC-UV that there were several additional minor EET-related peaks in the chromatograms. In the original study of monolayer oxidation (1), minor *trans*-monoepoxy products were formed from linoleic acid, so it was expected these additional peaks (peaks 3, 7, and 8) represented *trans*-epoxy EETs. To check this, peak 3 was isolated and first analyzed by GC-MS of the corresponding diol derivative (Material and methods). On GC, the peak 3 dihydroxyeicosatrienoic acid (methyl ester trimethylsilyl ether derivative) eluted immediately after the corresponding diol from the peak 2; their mass spectra were almost identical and in accord with a published spectrum (30), with a molecular ion at m/z 496 and prominent α-cleavage ions at m/z values of 173 (C_15_–C_20_), 275 (C_14_–C_20_), and 323 (C_1_–C_14_), thus establishing the peak 3 as a 14,15-EET isomer and confirming the analysis from LC-MS. Figure 4 illustrates the proton NMR spectra of the methyl esters of EET-2 and EET-3, respectively. These data were consistent with the proposed identifications as epoxy isomers of 14,15-EET, although close scrutiny of the key epoxide proton signals between 2.5 and 3 ppm was warranted to distinguish *cis* and *trans* epoxides. Unfortunately, the epoxide signals significantly overlap with the methylene protons of H7 and H10. To identify the most diagnostic solvent for distinguishing *cis* and *trans* 14,15-EETs, the proton spectra of the methyl esters of the peaks 2 and 3 were recorded in four different NMR solvents, d_6_-benzene, d-chloroform, d_4_-methanol, and d_3_-acetonitrile. The only epoxide proton to stand clear by itself is the H15 signal in d_6_-benzene; it appears as a double doublet of doublets with eight peaks that can be assigned three coupling constants (Fig. 5). Decoupling of protons on the adjacent carbons 13 and 16 (not shown) confirmed J_14,15_ = 4.2 Hz, which designates the *cis*-epoxide of 14,15-EET (peak 2). In the corresponding spectrum of peak 3 methyl ester in d_6_-benzene, the two epoxide protons stand clear of the methylene signals from H7 and H10, and although H14 and H15 partially overlap with one another, a ∼2 Hz coupling is evident on each side of the multiplet; decoupling of the signals from H13 and H16 confirmed J_14,15_ = 2.1 Hz, thus designating peak 3 as *trans*-14,15-EET.

The RP-HPLC chromatograms show the peaks of 15-HETE, 15-HPETE, 5-HETE, and 5-HPETE, labeled on Fig. 2B, C. These were identified from their retention times and characteristic UV spectra: 5- and 15-HETE give identical spectra (31), on our Agilent-diode array detector with λmax 236.6 nm; 15-HPETE and 5-HPETE give significantly different UV spectra, and identical to each other, λmax 237.3 nm. In Fig. 2B (60°C/45 min), the H(P)ETE signals in the 235 nm channel reached about half the absorbance value of the EET peaks 1 and 2 at 205 nm, although, on account of the intense absorbance of the conjugated dienes, the 235 nm absorbance signal biases the apparent abundance in favor of the H(P)ETEs. In the 37°C/6.5 h chromatogram (Fig. 2C), the HPETE peaks were more prominent than the HETEs, and their absorbance at 235 nm averaged slightly higher than the main EET peaks at 205 nm. Overall, the results support the prominence of EETs among the isolated monolayer-autoxidation products.

## Discussion

### EETs as autoxidation products

The relative prominence of EETs in the monolayer autoxidations is quite striking and in complete contrast to the typical autoxidation of arachidonic acid. To help add potential biological significance to the findings, we compared the reactions at 60°C with 37°C, producing similar results at each temperature. The monolayer autoxidations yielded approximately similar amounts of the four EETs, although 5,6-EET was represented almost entirely as the 5-δ-lactone, a facile transformation that appears to be catalyzed by further interactions with the silica. Interestingly, this lactone derivative of 5,6-EET is proposed as a potential endothelial cell-derived relaxing factor (32). The structures of the 5-δ-lactone and representative *cis*- and (minor) *trans*-epoxides (14,15-EETs) were confirmed by ^1^H-NMR. The proton NMR data on EET-related fatty acid mono-epoxides are in the literature, although tabulated as a list of ppm values with most signals designated as multiplets, which provides a minimal basis for a detailed comparison with laboratory data. Our results show the appearance of the signals, which can be helpful for comparison with future work.

### Mechanistic considerations

**Scheme 1:**
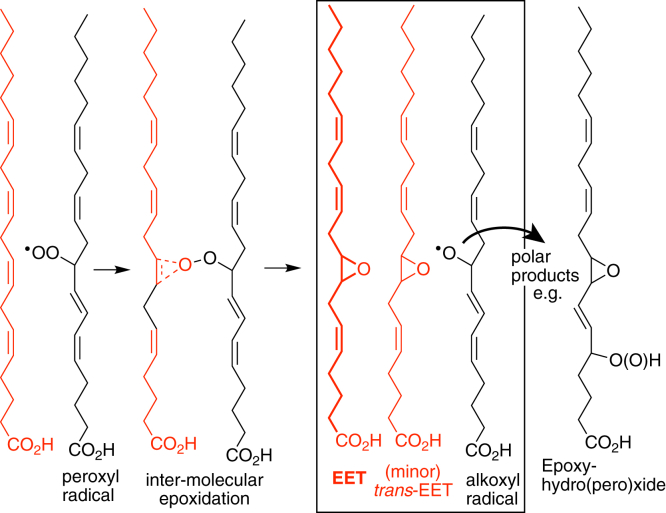
Mechanism of epoxyeicosatrienoic acids formation in monolayer autoxidation.

As deduced in the original papers with oxidation of linoleic acid, the fatty acid epoxidations occur by inter-molecular reaction between polyunsaturated fatty acid molecules aligned in the monolayer (1, 6). The proposed mechanism is illustrated in Scheme 1. Of note, it is part-and-parcel of this mechanism that *a*) the initiating species is a fatty acid peroxyl, produced by the classic mechanism of lipid peroxidation that normally accumulates as fatty acid hydroperoxides, *b*) in the course of the inter-molecular interaction, a small proportion of the recipient carbon chains swivel, leading to the formation of a *trans*-epoxide, (and *trans*-EETs occur naturally as discussed in the last section on biological significance), and *c*) for every fatty acid monoepoxide produced, the reaction yields a highly reactive alkoxyl radical (33), which will spark further transformations and synthesis of the polar products. These include epoxy-hydro(pero)xides (illustrated in Scheme 1) or chain cleavage products and endoperoxides as discussed further below. These polar derivatives are evident as a streak of polar TLC spots in the originally reported monolayer oxidation of linoleate (1), and in the Fig. 2 chromatograms as the complex mixture of early-eluting oxidative derivatives detected with 205 nm absorbance.

### Recovery of H(P)ETEs

An interesting and mechanistically significant point is raised by the isolation of only 5- and 15-H(P)ETEs and absence of the other four arachidonate hydro(pero)xides (at C8, C9, C11, and C12). This phenomenon, of the relative prominence of the 5 and 15 hydroperoxides from the autoxidation of arachidonic acid, was reported by Fridovich and Porter back in 1981 (34). It is well established that there is no positional preference to the initial nonenzymatic oxygenation (35), and therefore the preponderance of 5 and 15 products does not signify a bias toward oxygenation at the 5 and 15 carbons. Instead, it is a reflection of the greater reactivity of the “internal” peroxyl radicals (at C8, C9, C11, and C12), which readily undergo intramolecular reaction with the adjacent (β position) double bond to form endoperoxides (4, 36, 37), or they more readily undergo carbon chain cleavage (38). (In Fig. 2, the small peak in between the 5- and 15-H(P)ETEs at 11.5 to 12 min is not an H(P)ETE, it is a mixture of two endoperoxide-hydroperoxides) (36). These outcomes are unavailable (i.e., endoperoxide formation) or at least less facile (for chain cleavage) for the 5- and 15-peroxyl radicals, so it is their comparative lack of reactivity that accounts for their relative overabundance among the recovered autoxidation products.

### Biological significance

The extent to which nonenzymatic EET formation could occur in cell membranes is an open issue. The enzymatic synthesis of EETs is catalyzed by cytochromes P450 and most large esters are not P450 substrates, lyso-phospholipids being an exception (39). Despite the lack of direct enzymatic epoxidation in cell membranes, there is unequivocal evidence for P450 products being incorporated into liver and kidney cell membranes: not only do the chiralities of these membrane-esterified EETs signify at least partial enzymatic origin (nonenzymatic products being racemic) (20), there is a further confirmation from the changes in the chiral profiles with the induction of different P450 enzymes (40). Remarkably, the membrane-incorporated EETs can modulate various bioactivities, from endothelial cell-dependent vascular relaxation, to L-type calcium channel activity, to glucose homeostasis (41, 42, 43), so their occurrence is potentially significant. Furthermore, the oxidized lipids including epoxy-fatty acids can be preferentially cleaved from esterification in cell membranes (e.g., refs (44, 45, 46, 47)), and this is recognized as a source of EETs in plasma (48, 49, 50, 51).

One of the issues, and a significant mechanistic twist to the monolayer epoxidations compared with P450-catalyzed transformations, is the coproduction of *trans*-EETs as we have demonstrated. The natural occurrence of *trans*-EETs in red cell membranes and phospholipids is well documented and is a source of these free lipids in animal and human blood plasma (48, 52, 53). In the spontaneously hypertensive rat, *trans*-14,15-EET is a more potent vasodilator than its *cis* counterpart and it is also preferentially hydrolyzed (inactivated) by soluble epoxide hydrolase, leading to a reduction of plasma *trans*-EETs being implicated in the increased blood pressure (50). The origin of these *trans*-EETs is matter of speculation (54) and the participation of monolayer-type epoxidation merits consideration.

Regarding the issue of yield, in the monolayer experiments, the inter-molecular oxidations that form EETs should be more prominent than in cell membranes with their mixtures of saturated and unsaturated lipids, although the anticipated lower production in the cell environment still might be compatible with the low percentage of esterified EETs in cells. In realistic terms, the direct nonenzymic epoxidation of membrane lipids is most likely associated with the strong conditions of lipid peroxidation, because, as noted above, the formation of epoxides by intermolecular reaction of a peroxyl radical produces in parallel an alkoxyl radical from which will be derived further highly reactive lipid-peroxidizing species. Thus, intense lipid peroxidation might be a condition associated with the “monolayer”-type epoxidation of membrane lipids.

## Data availability

Data are contained in the article and as supplemental Tables S1 and S2.

## Supplemental data

This article contains supplemental data.

## Conflict of interest

The authors declare that they have no conflicts of interest with the contents of this article.
