# Supplementary material for: Monolayer autoxidation of arachidonic acid to epoxyeicosatrienoic acids as a model of their potential formation in cell membranes
Source: J Lipid Res. 2021 Dec 2;63(1):100159. doi: 10.1016/j.jlr.2021.100159 (PMC8953651; doi:10.1016/j.jlr.2021.100159)
Supplement: Supplemental Tables S1 and S2 [file mmc1.docx]

**Supplemental data**

**Monolayer autoxidation of arachidonic acid to epoxyeicosatrienoic acids as a model of their potential formation in cell membranes**

By James A. Weiny_,_ William E. Boeglin, M. Wade Calcutt_,_ Donald F. Stec and Alan R. Brash

**Table S1. ^1^H NMR (600 MHz) chemical shifts (to the nearest 0.01 ppm) and coupling constants for EET-1, 5-δ-lactone, 6-hydroxy-eicosatrienoate in *d6*-benzene**

| Chemical shift  (ppm) | Multi-plicity | Proton position  (Carbon  No.) | Number  of  protons | Coupling constants (Hz) |
| --- | --- | --- | --- | --- |
| 5.55-5.42 | m | 8,9,11,  12,14,15 | 6 |  |
| 3.63-3.60 | dt | 5 | 1 | J_5,6a,b_ = 3.6  J_6,7_ = 11.4 |
| 3.19 | m | 6 | 1 |  |
| 2.91-2.89 | t | 10 or 13 | 2 | J ≈ 6 |
| 2.88-2.86 | t | 10 or 13 | 2 | J ≈ 6 |
| 2.21 | m | 7 | 2 |  |
| 2.075 | q | 16 | 2 | J ≈ 6 |
| 2.016 | m | 2 | 1 |  |
| 1.91 | ddd | 2 | 1 | J ≈ 7 and 9.5 |
| 1.70 | d | -OH | 1 | J_6,OH_ = 6.6 |
| 1.34 | m | 18 | 2 |  |
| 1.26 | m | 19,17 | 4 |  |
| 1.20 | m | 4a | 1 |  |
| 1.10 | m | 3a | 1 |  |
| 1.40 | m | 4b | 1 |  |
| 0.96 | m | 3b | 1 |  |
| 0.88 | t | 20 | 3 | J ≈ 7 |

**Table S1. ^1^H NMR (600 MHz) chemical shifts (to the nearest 0.01 ppm) and coupling constants**

**for EET-2 (14,15-*cis*-EET) and EET-3 (14,15-*trans*-EET) in *d6*-benzene**

| Chemical shift  (ppm) | | Multiplicity | | Proton position  (Carbon  No.) | | Number  of  protons | | Coupling constants  (Hz) | |
| --- | --- | --- | --- | --- | --- | --- | --- | --- | --- |
| EET-2  14,15-*cis*-EET | EET-3  14,15-*trans*-EET | EET-2 | EET-3 | EET-2 | EET-3 | EET-2 | EET-3 | EET-2 | EET-3 |
| 5.54-5.46 | 5.54-5.48 | m | m | 8,12 | 8,12 | 2 | 2 |  |  |
| 5.44-5.38 | 5.45-5.38 | m | m | 6,9,11 | 6,9,11 | 3 | 3 |  |  |
| 5.29 | 5.29 | m  (*cis*) | m  (*cis*) | 5 | 5 | 1 | 1 |  |  |
| 3.345 | 3.345 | s | s | OMe | OMe | 3 | 3 |  |  |
| 2.83-2.77 | 2.82-2.77 | m | m | 7,10,14 | 7,10 | 5 | 4 |  |  |
| 2.715 | 2.56 | ddd | m | 15 | 14,15 | 1 | 2 | J_14,15_ = 4.2  J_15,16a or 16b_ 5.1 and 6.8 | (J_14,15_ = 2.1) |
| 2.36 | 2.32 | m | m | 13a | 13a | 1 | 1 |  |  |
| 2.16 | 2.19 | m | m | 5 | 13b | 1 | 1 |  |  |
| 2.10 | 2.10 | t | t | 2 | 2 | 2 | 2 | J_2,3_ = 7.4 | J_2,3_ = 7.4 |
| 1.98 | 1.98 | q | q | 4 | 4 | 2 | 2 | J_4,5_ = 7.4 | J_4,5_ = 7.4 |
| 1.60 | 1.60 | p | p | 3 | 3 | 2 | 2 | J_2,3_ = J_3,4_ = 7.4 | J_2,3_ = J_3,4_ = 7.4 |
| 1.45 | 1.41 | m | m | 16a | 16a | 1 | 1 |  |  |
| 1.38 | 1.35 | m | m | 16b,17a | 16b,17a | 2 | 2 |  |  |
| 1.30 | 1.29 | m | m | 17b | 17b | 1 | 1 |  |  |
| 1.21 | 1.20 | m | m | 18,19 | 18,19 | 4 | 4 |  |  |
| 0.86 | 0.85 | t | t | 20 | 20 | 3 | 3 | J_19,20_ = 7.0 | J_19,20_ = 7.0 |
